# An Introduction to Advanced Targeted Acquisition Methods

**DOI:** 10.1016/j.mcpro.2021.100165

**Published:** 2021-10-18

**Authors:** Mirjam van Bentum, Matthias Selbach

**Affiliations:** 1 Proteome Dynamics, Max Delbrück Center for Molecular Medicine, Berlin, Germany; 2 Charité-Universitätsmedizin Berlin, Berlin, Germany

**Keywords:** targeted proteomics, PRM, SRM, MRM, Picky, iRT, MaxQuant.Live, IS-PRM, SureQuant, TOMAHAQ, DDA, data-dependent acquisition, DIA, data-independent acquisition, iRT, indexed retention time, LOD, limit of detection, LOQ, limit of quantification, maxIT, maximum injection time or fill time, MRM, multiple reaction monitoring, PRM, parallel reaction monitoring, QqOrbi, quadrupole-Orbitrap, QqQ, triple quadrupole mass spectrometer, QqTOF, quadrupole-TOF, RT, retention time, SIL, stable isotope labeled, SILAC, stable isotope labeling by amino acids in cell culture, SRM, selected reaction monitoring, TMT, tandem mass tag, TOMAHAQ, triggered-by-offset, multiplexed, accurate-mass, high-resolution, and absolute quantification

## Abstract

Targeted proteomics *via* selected reaction monitoring (SRM) or parallel reaction monitoring (PRM) enables fast and sensitive detection of a preselected set of target peptides. However, the number of peptides that can be monitored in conventional targeting methods is usually rather small. Recently, a series of methods has been described that employ intelligent acquisition strategies to increase the efficiency of mass spectrometers to detect target peptides. These methods are based on one of two strategies. First, retention time adjustment-based methods enable intelligent scheduling of target peptide retention times. These include Picky, iRT, as well as spike-in free real-time adjustment methods such as MaxQuant.Live. Second, in spike-in triggered acquisition methods such as SureQuant, Pseudo-PRM, TOMAHAQ, and Scout-MRM, targeted scans are initiated by abundant labeled synthetic peptides added to samples before the run. Both strategies enable the mass spectrometer to better focus data acquisition time on target peptides. This either enables more sensitive detection or a higher number of targets per run. Here, we provide an overview of available advanced targeting methods and highlight their intrinsic strengths and weaknesses and compatibility with specific experimental setups. Our goal is to provide a basic introduction to advanced targeting methods for people starting to work in this field.

## Highlights

• Advanced acquisition methods improve focus of mass spectrometers on target peptides.
• This review discusses existing methods based on two strategies.
• *Retention time adjustment-based* methods enable intelligent scheduling of peptide RTs.
• In *spike-in triggered acquisition* methods targeted scans are initiated by spike-ins.

## Graphical abstract

**Figure:**
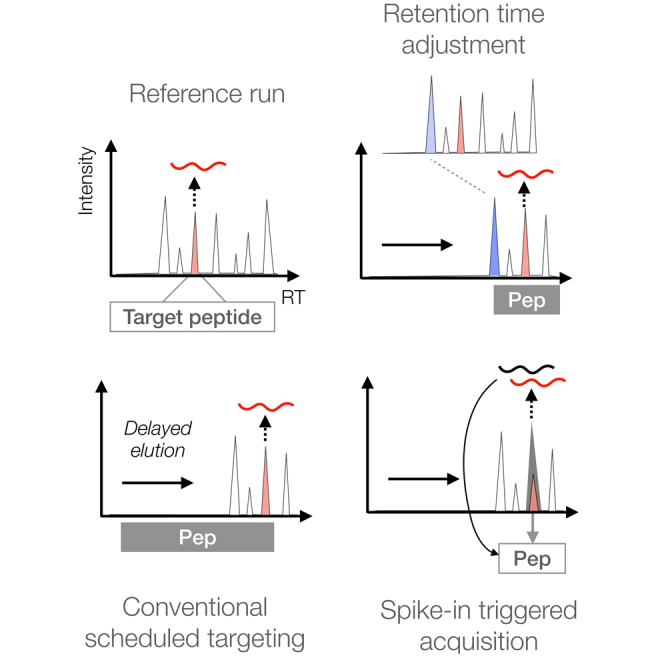

## In Brief

The analytical power of targeted proteomics depends on how efficiently the mass spectrometer detects target peptides. A number of “smart” acquisition approaches have been developed that enable more targets per run and improve analytical performance such as sensitivity, specificity, and quantitative accuracy. This review provides an introduction to these methods and highlights their inherent strengths and weaknesses.

In mass spectrometry (MS)-based proteomics, data acquisition strategies can be broadly characterized as discovery or targeted approaches. Discovery approaches such as data-dependent acquisition (DDA) and data-independent acquisition (DIA) aim to maximize the number of peptide identifications/quantifications per measurement time (1, 2). Since DDA and DIA are biased toward detection of more abundant peptides, both of these discovery approaches have drawbacks when it comes to the reproducible identification and quantification of specific low-abundance peptides. As many peptides of special interest are often of low abundance (such as peptides derived from proteins thought to be particularly “relevant” such as kinases, transcription factors, or specific posttranslationally modified peptides), this limits the broad application of discovery methods in high-throughput basic research or clinical setups (3, 4, 5, 6, 7, 8, 9, 10).

Targeted approaches such as selected reaction monitoring (SRM, also known as multiple reaction monitoring or MRM) (11, 12) and parallel reaction monitoring (PRM) (13, 14) enable highly sensitive, reproducible, and fast detection and accurate quantification of predefined sets of target peptides. For detailed information on these technologies, we refer the reader to excellent reviews (15, 16, 17). Briefly, during a standard SRM or PRM run, the mass spectrometer continuously acquires spectra at the expected mass to charge (m/z) ratio and chromatographic retention time (RT) of target peptides. SRM experiments are performed on triple quadrupole mass spectrometers where precursor ions are selected in the first quadrupole and fragmented in the second quadrupole. Target-specific fragment ions are then selected in the third quadrupole for detection. Typically, several fragment ions are successively monitored. Triple quadrupole mass spectrometers are robust and relatively cheap, contributing to implementation of SRM assays in a clinical setting (9, 18). In contrast to SRM, PRM experiments are performed on systems able to record whole fragment spectra such as Quadrupole-Orbitrap type mass spectrometers and QqTOF Systems. In PRM, all fragment ions of a selected precursor are measured in parallel. In conventional SRM or PRM set ups, an inclusion list is passed on to the mass spectrometer that dictates the precursor m/z ratio windows and RT windows. On specific systems more scan parameters can be included such as scan-specific fragmentation energies or injection times. For SRM, the inclusion list additionally specifies the m/z of the fragment ions to be monitored.

The selection of the suitable target peptides and fragment ions as surrogates for the target proteins is essential to the sensitivity, specificity, and analytical power of targeted assays (17, 19, 20). But this can be laborious and time-consuming: Peptides need to be selected considering their proteotypicity and detectability (21) and for the fragment ions that are monitored in SRM assays, the analytical specificity (low signal interference) is very important. This can be a bottleneck for certain applications of conventional targeted methods due to the time-consuming nature of the evaluation and validation process (22). To facilitate this process, libraries with empirically determined peptide fragmentation spectra have been established (23, 24). Alternatively, fragmentation spectra can also be predicted *in silico* (25, 26). Resources such as the online database SRMAtlas (23), Skyline software (27) and others (28, 29, 30) assist in the design of targeted methods by facilitating access to online repositories of empirical data, enabling *in silico* prediction of spectra or validation of transitions.

Key advantages of targeted approaches over discovery approaches are that they allow highly specific and sensitive measurements (17). Specificity results from the two successive mass filtering steps—one at the precursor ion (MS1) and one at the fragment ion (MS2) level, which greatly increases the signal-to-noise ratio. Sensitivity depends on the time the mass spectrometer spends on collecting target peptide ions, the so-called dwell time. During targeted acquisition, the mass spectrometer only needs to analyze target peptides and can ignore all others, which results in longer dwell times compared with discovery approaches. In SRM, the dwell time per transition can highly vary between and within setups. Ranges from 13 to 256 ms have been reported (31, 32) (Table 1). Typically, multiple transitions per peptide are monitored. In PRM, where the whole peptide spectrum is acquired at once, reported ion accumulation times vary from 15 to 550 ms (Table 1). In combination, the high specificity and sensitivity of targeted methods allow analysis of peptides that escape detection in discovery experiments (12, 13).

**Table 1:** Representative methods from literature

Scan type	MS system	Instrument	Gradient length (min)	Peptides	# Transitions monitored	Detection window size	Average peak width	Points per peak	Dwell time	Cycle time	Reported sensitivity	Year, reference
SRM	QqQ-ion trap	4000QTrap (ABI/MDS-Sciex)	30–60	N.R.	30–60	3 m	3.5 s	N.R.	20–200 ms	N.R.	39 copies/cell	2009 (31)
SRM	QqQ	Xevo TQ-S Triple Quadrupole mass spectrometer (Waters, Milford, MA)	180	800	2400	8	1 m	20	8–107 ms	N.R.	N.R.	2014 (42)
SRM	QqQ	Xevo TQ-S Triple Quadrupole mass spectrometer (Waters, Milford, MA)	180	82	246	8	35 ms	20	29–142 ms	N.R.	Median LOQ 20.2 ng/ml in 2 μg background	2014 (42)
SRM	QqQ	6490 triple quadrupole mass spectrometer (Agilent Technologies)	38	350	1050	1	N.R.	N.R	13–256 ms	515 ms	6 ng/ml, 261 μg/ml	2014 (32)
SRM	QqQ	5500 QTRAP mass spectrometer (ABSciex)	37	324	972	2 m 30 s	N.R.	N.R.	N.R.	1.5 s	Median LLOQ 50 fmol/μg in 1.0 μg/μl background	2014 (88)
SRM	QqQ	Quantiva triple quadrupole mass spectrometer (Thermo Fisher Scientific)	48.5	97	388	2 m 30 s	1.5 s	N.R.	N.R.	N.R.	LOQ: 80–8000 fm/mg	Direct-MRM, 2021 (18)
PRM	QqOT	Q-Exactive mass spectrometer (Thermo Fisher Scientific)	22	64	N.A.	3 m	N.R.	N.R	maxIT: 30 ms	60–900 ms	Similar to SRM (same study)	2015 (40)
SRM	QqQ	TSQ Vantage Triple Stage Quadrupole Mass Spectrometer (Thermo Fisher Scientific)	22	42	124	3 m	N.R.	N.R	15 ms	90–540 ms	Similar to PRM (same study)	2015 (40)
PRM	QqOT	Orbitrap Fusion Lumos Tribrid Mass Spectrometer (Thermo Fisher Scientific)	1 h 15 m	21	N.A.	2 m	N.R.	N.R.	maxIT: 500 ms	3 s	4–103 amol	2019 (41)
PRM	QqOT	Q-Exactive mass spectrometer (Thermo Fisher Scientific)	60	25	N.A.	5–10 m	N.R.	N.R.	maxIT: 120 ms	3.25 s	N.R.	2012 (13)
SRM	QqQ	TSQ Quantum Discovery Max (Thermo Fisher Scientific)	60	14	42	7–60 m	N.R.	N.R.	35 ms	0.63 s	N.R.	2012 (13)
PRM	QqOT	Q Exactive Plus (Thermo Fisher Scientific)	360 min (200 cm column)	1067	N.A.	3.8–8 m	30 s	5	maxIt: 190 ms	6 s	N.R.	2017 (89)
PRM	QqTOF	TripleTOF 5600 (QqTOF) mass spectrometer	65	15	N.A.	Unscheduled	N.R.	10–15	maxIT: 150 ms	2.55 s	Mean LOD: 153 amol/μl in 1 μg/μl background	2015 (90)
PRM	QqTOF	TripleTOF 5600 (QqTOF) mass spectrometer	90	532	N.A.	2 m	N.R.	10–15	maxIT: 60 ms	3.3 s	N.R.	2015 (90)

In publications where multiple methods were tested, representative methods were selected for the table.

Abbreviations: maxIT, maximum injection time or fill time; QqQ, Triple quadrupole Mass Spectrometer; QqOT, Quadrupole-Orbitrap; QqTOF, quadrupole time-of flight.

In addition, accurate (and potentially absolute) quantification can be achieved using stable isotope labeled (SIL) spike-in peptides as internal standards (33, 34). Monitoring both heavy and the light peptides can then be used to quantify the abundance of endogenous peptides relative to the SIL spike-in. Alternatively, quantification can also be done “label-free” or by spiking in a labeled reference peptide (LRP) (35, 36, 37). SRM has early on been shown to reach a limit of detection (LOD) in the attomole range (17, 38), and PRM performs similarly in terms of sensitivity, dynamic range, and quantitative accuracy (39, 40) (Table 1). In a recent publication, PRM reached a limit of quantification (LOQ) ranging from 4 amol to 103 amol depending on the target peptide (41).

A major drawback of targeting approaches is the limited number of peptides that can be targeted in parallel: Typical PRM-based methods enable reliable detection and quantification of 10 to 100 target peptides (5) (Table 1), but when the number of target peptides increases, the performance suffers. SRM methods have already quantified up to 100 s of peptides in a single LC run (42) (Table 1). However, this requires optimized chromatographic conditions and tedious selection of specific transitions. In recent years, a range of new tools and methods have been developed that use intelligent acquisition strategies to allow the mass spectrometer to better focus on the targeted peptides by intelligent scheduling strategies. In this review we discuss these *advanced acquisition methods* and how they can improve the analytical performance and simplify targeted method design.

In general, the number of peptides that can be targeted per run depends on several factors. First, since the mass spectrometer can only target a single peptide at any given time, the dwell times needed for sensitive detection determine the number of peptides that can be monitored in parallel. The limit of detection results from the interplay between the expected width of elution peaks, the number of points across the peak necessary for quantification (typically 8–15 points (43, 44)), the dwell time per peptide or transition, the number of peptides and transitions (for SRM) looked at in parallel, and the MS cycle time. Targeting too many peptides at the same RT results in long MS cycle times, which reduces the number of data points acquired across elution profiles and has detrimental consequences on quantification and reliability of detection. Similarly, reducing the dwell time, and thereby cycle time, leads to decreased sensitivity.

**Fig. 1:**
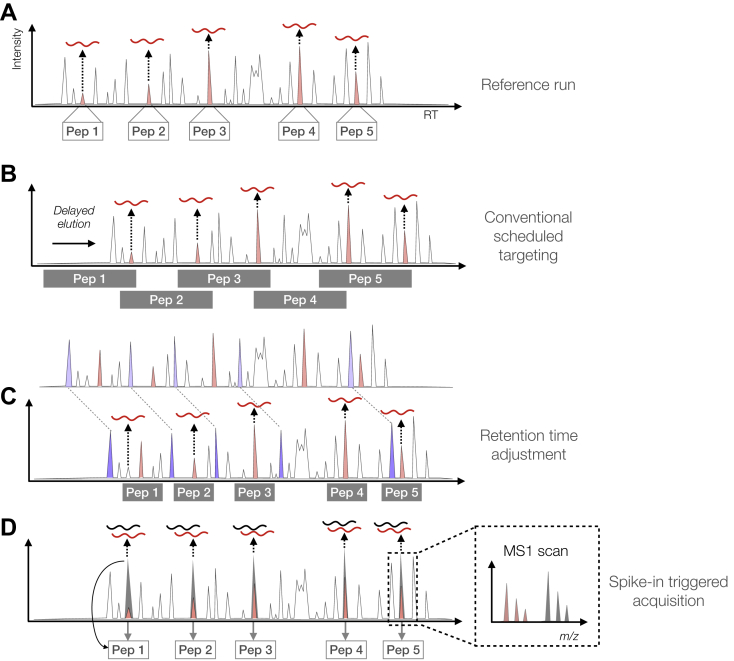
**Advanced targetedacquisitionmethods.** Schematic elution profiles with target peptides (in *red*) are shown. *Gray filled boxes* depict monitoring windows in which continuous targeted scans are performed. *A*, elution profile of the reference run. Target peptide elution peaks are indicated in *red*. Elution profiles in (*B*), (*C*), and (*D*) depict three different strategies to compensate for RT shifts (in this case delayed relative to the reference run). *B*, conventional targeting methods use wide monitoring windows (centered on expected elution time) to ensure that peptides are detected despite RT shifts. *C*, RT prediction methods enable advanced targeting by better adjusting peptide RTs before (offline) or during (online) targeted acquisition. *Blue* “iRT” peptides (or background peptides) are used to estimate shifts from predicted or previously observed RT and shift the monitoring windows accordingly. This results in shorter monitoring windows. *D*, spike-in-triggered acquisition methods rely on synthetic spike-in peptides (*gray*). Detection of these peptides by the mass spectrometer triggers acquisition of corresponding endogenous target peptides.

By scheduling targeted experiments, the number of target peptides can be increased. In contrast to unscheduled targeted experiments, where the same peptides are monitored throughout the entire run, in scheduled targeting experiments peptides are only targeted during a monitoring window around their expected RT. These monitoring windows are a second factor that limits the number of targets. To ensure reproducible detection, monitoring windows need to be wide enough to capture deviations from the expected elution time (Fig. 1, *A* and *B*). The windows are typically about five to ten times wider than actual chromatographic peak widths (depending on the gradient length, see Table 1), which decreases the number of peptides that can be targeted per run.

Several *advanced acquisition methods* have been developed in recent years that address these challenges by intelligent scheduling schemes. In general, these advanced targeting methods employ one of two strategies. The first strategy is *retention time adjustment*, which is a variation of conventional scheduled targeted acquisition. In these methods, the aim is to keep the targeting windows shorter by adjusting the expected retention time to achieve a more accurate estimation of the actual retention time (Fig. 1*C*). The second strategy does not make use of targeting windows, but instead uses synthetic spike-in reference peptides to *directly trigger acquisition* of endogenous target peptides (Fig. 1*D*).

The available advanced acquisition methods are based on different ideas and have been established on different mass spectrometers. This makes it difficult to choose a method that is well-suited for a specific application. Also, we realized that many original papers describing advanced acquisition methods do not reference each other, which makes it difficult for new scientists in the field to get an overview of the methods currently available. Here, we provide a basic overview of currently available advanced acquisition methods for targeted runs. Rather than reviewing these methods in detail, we focus on their conceptual differences and highlight their inherent strengths and weaknesses. A summary of the methods discussed here is provided in Table 2, aligned by their underlying strategies.

**Table 2:** Technical overview of advanced targeting methods mentioned in review, with key analytic parameters of application examples

Method	Machine compatibility	Quantification	# Of targets	Sensitivity	Ease of implementation	Reference	Real time	Demonstrated applications	
Retention time adjustment								Application examples	Specifics
Picky	Thermo Q Exactive, Exploris, Fusion (Lumos), Agilent TOF	SIL, label-free	+	+	+++	Calibration file, ProteomeTools	N	Technical benchmarking (51), cross-validation discovery data (91)	Q Exactive HF-X (Thermo Fisher Scientific) 40 min gradient 80% of peptide-RT predicted in a 1.9 min window (online updated version)
iRT (‘offline’)	N.A.	SIL, label-free	+	+	++	Reference run of set of standard spike-in peptides	N	A.o. Technical benchmarking (52), Proteomic characterisation of COVID-19 patient sera (92), immunotherapy mutant epitope detection (93)	TSQ Vantage Triple Quadrupole, 90 min gradient 148 peptides, 1232 transitions 4× more precise then SSRCalc (from 21.9 min to 5.1 min) (52)
iRT (‘on-the-fly’)	a.o. Thermo Q Exactive HF-X	label-free	+	+	++	Set of standard spike-in peptides	Y	dRT technical benchmarking	Q Exactive HF-X (Thermo Fisher Scientific) ∼40 min gradient (varied) 198 peptides targeted maxIT 40 ms Reduction window size from 3 to 1 min
MaxQuant.Live	Thermo Orbitrap (Tunefile 2.9 or higher)	SIL, label-free	++	+	+	Reference run, MS1	Y	Technical benchmarking (56)	Q Exactive HF-X (Thermo Fisher Scientific) 100 min gradient 20,000 peptides maxIT 110 ms Reduction window size from several minutes to <1 min
Remes *et al.*	Modified Orbitrap Fusion Lumos	N.A.	++	+	N.A.	Reference run, DIA MS/MS	Y	Technical benchmarking (57)	Modified Orbitrap Fusion Lumos (Thermo Fisher Scientific) 60 min gradient 1489 targets maxIT 13 ms, cycle time 1.7 s 300% reduction of targets monitored parallel

Spike-in triggered acquisition						Trigger	Spectral match		
SureQuant	Thermo Exploris 480	SIL, label-free	+++	++	+	SIL-peptide	Y	pTyr signalling networks in cancer (62), absolute quantification of peptide major histocompatibility complex antigens (94)	Orbitrap Exploris 480 (Thermo Fisher Scientific) 60 min gradient 340 target peptides 116–244 ms maxIT
Pseudo-PRM	Thermo Q Exactive	SIL, label-free	++	+	+	SIL-peptide	N	Technical benchmarking (63)	Q Exactive Plus (Thermo Fisher Scientific) 200 cm column, 140 min gradient maxIT 50 ms 1080 target peptides
TOMAHAQ	Tribrid systems	TMT	+++	+++	++	TMTsh peptide	Y	Metabolism- and inflammation pathways in aging (64)	Orbitrap Fusion Lumos (Thermo Fisher Scientific) 180 min gradient 520 target peptides maxIT 5000 ms
Scout-MRM	Sciex instruments	SIL, label-free	+	+	++	Scout peptide	N	Protein expression in plant infection (68), multi residue pesticide monitoring (95)	QTRAP 6500 (Sciex) 120 min gradient Target 782 peptides, 2346 transitions Dwell time: min 5 ms Target scan time: 1.2 s

Abbreviations: a.o., among others; N.A., not applicable; N.R., not reported; SIL, stable isotope labeled.

## Retention Time Adjustment-Based Methods

Retention time (RT) adjustment methods estimate the RTs of target peptides and adjust monitoring windows accordingly. More accurate estimation of peptide RTs means that monitoring windows can be kept shorter without missing peptides, thus enabling more target peptides per run (Fig. 1*C*).

RTs are not universal constants for a given peptide but are affected by the specific chromatographic conditions employed (type, length, and age of the column, buffer composition, LC gradient, flow rate, etc). In a conventional scheduled targeting experiment, target peptide RTs for a specific setup are either predicted or determined empirically in measurements prior to the experiment. Prediction of retention time is either based on intrinsic peptide properties such as hydrophobicity (45, 46), or on machine learning approaches (47, 48) deep learning (25, 49). To facilitate design of scheduled targeted experiments, the prediction tools SSRCalc and Prosit are implemented in Skyline (27, 45, 46). However, the prediction of peptide RT is imperfect, and research on RT prediction for posttranslationally modified peptides has been lacking (50). Empirically determining peptide RTs can be costly (when a peptide needs to be synthesized) and time-consuming. In addition, even when RTs have been correctly determined or predicted, they still vary stochastically from run to run (due to variability in LC pump speed, sample pick-up volume, changes in background, etc.).

RT adjustment-based methods aim to improve the accuracy of prediction “offline” as well as “on-the-fly.” Static RT adjustment methods that employ *indexed Retention Times* (“offline” iRT-based methods) or *Picky* estimate RTs of target peptide “offline” (that is, before the targeted acquisition run) and can therefore adjust RT shifts that arise due to user-specific chromatographic conditions. Real-time adjustment methods use spike-in standards (*“on-the-fly” iRT-based methods*) or background peptides (*spike-in free real-time adjustment*) to adjust monitoring windows in real time (that is, during the targeted acquisition run). The latter approaches are therefore also robust against run-to-run variability.

### Picky

Picky is an online tool that simplifies the design of PRM and SRM experiments and adjusts peptide RTs to a specific HPLC setup (51). To this end, the tool uses experimentally determined peptide RTs and fragmentation spectra from the ProteomeTools database (24). The main input from the user is simply a list of human (or mouse) proteins to be targeted. Based on this input, Picky selects corresponding high scoring target peptides from the ProteomeTools database together with their experimentally observed RTs and suitable collision energies. To adjust RTs to specific HPLC conditions, users can upload any list of peptides and RTs generated on their own chromatographic setup. Picky then uses this information to rescale RTs of target peptides to the user-specific HPLC conditions. This significantly reduces RT-shifts based on LC-characteristics, thus enabling narrow monitoring windows. The tool exports optimized PRM/SRM acquisition methods in formats compatible with different mass spectrometers. Picky also exports a spectral library of target peptides that are useful for validation of the PRM/SRM data obtained. The main advantages of *Picky* are that it makes setting up optimized targeted acquisition methods easy and that the tool is compatible with different machines. A disadvantage is that the static RT rescaling performed by *Picky* cannot capture run-to-run variability.

### Indexed Retention Times (iRTs)

Indexed retention times (iRTs) are based on the idea to spike-in a standard set of reference peptides in order to normalize RTs of peptides in the sample (52). The iRT of a peptide is a dimensionless number representing the RT of a peptide relative to the reference peptides. By converting actual RTs to a reference-based index, RTs can be adjusted to different chromatographic set-ups. Similar to *Picky*, this can be used to transfer static RT estimates across labs and methods (“offline” iRT-based methods). A major advantage of the iRT concept is that it is widely integrated into publicly available databases and tools such as ProteomeTools (24), SRMatlas (23) and Prosit (25). Also, Skyline facilitates the setup of scheduled targeted run design using peptide iRT values derived from empirical data, databases, or the predicted iRT values from Prosit (27, 53).

In addition, iRTs can also be used to estimate target peptide RTs “on-the-fly” (real-time adjustment of expected RTs) *via* dynamic RT window correction on some mass spectrometers (for example, over the *dynamic Retention Time* feature on Thermo HF-X instruments). The real-time adjustment of RTs reduces run-to-run variability. The on-the-fly adjustment of target windows was first shown using a mixture of 150 synthetic isotopically labeled peptides (54). While any custom set of standard reference peptides can be used to define an iRT scale, the “iRT kit” from Biognosys, the “Peptide Retention Time Calibration Mixture” from Pierce, and “PROCAL” from JPT (55) are three commercially available options.

### Spike-in Free Real-time Adjustment

While iRTs require spiking-in synthetic reference peptides, other methods take advantage of endogenous background peptides to estimate RTs of target peptides in real time. MaxQuant.Live is a software framework for real-time monitoring of mass spectrometric data and controlling data acquisition (56). One of the applications implemented in this framework is “global targeting,” which leverages 10,000 easily recognizable peptides from a reference run to continuously minimize the median differences between the observed and the expected RTs. This adaptive nonlinear RT adjustment shrinked monitoring windows to less than 1 min and enabled recognition and targeting of more than 25,000 peptides in single LC-MS runs. The MaxQuant.Live framework is flexible and allows users to define target peptide-specific dwell times, sequential isolation of light and heavy peptides, or cofragmentation of light and heavy peptides together. The software is freely available and compatible with Thermo Orbitrap mass spectrometers (tune version 2.9 or higher).

Where the Maxquant.Live approach uses MS1 scans to monitor RT shifts, an alternative real-time chromatographic alignment approach from the MacCoss lab uses MS2 survey scans instead (57). To this end, the mass spectrometer periodically acquires DIA MS2 survey scans. These MS2 scans are aligned to a reference DIA experiment in real time in order to determine the RT shift and to adjust the active target list accordingly. With this strategy, the authors were able to reduce the peptide monitoring windows from 5 min to 1 min in a 60 min gradient. Acquiring these data at the MS2 rather than at the MS1 level increased robustness and selectivity of the RT alignment. The alignment was shown to be rather robust against changes in background abundance by performing dilution experiments. The method was implemented on a modified Fusion Lumos and is not yet freely available.

Key advantages of spike-in free real-time acquisition methods are that they do not require spike-in peptides, and the high number of datapoints leveraged by the algorithms facilitates highly precise estimates of target peptides RT. These methods can take full advantage of the data while it is still being acquired by using complex data-dependent decision trees. However, these methods are rather complex and depend on sufficient overlap in identified peptides between the measured sample and the reference library.

## Spike-in-Triggered Acquisition Methods

The methods listed in the previous section aim to improve the precision of RT estimates, thereby enabling narrower targeting windows. The targeting windows, however, will most likely still exceed the elution peak of the target peptides, thereby reducing the number of targets per run. Methods based on spike-in-triggered acquisition circumvent peptide RT estimation and targeting windows all together. Instead of acquiring data at specific (adjusted) predefined RTs, the acquisition is initiated by the detection of trigger peptides. To this end, a synthetic heavy stable isotope labeled (SIL) peptide is added to the sample for every endogenous target peptide. During spike-in-triggered acquisition, the mass spectrometer switches between two types of scanning modes: a “watch" mode and a “quantification" mode. In the watch mode, the mass spectrometer scans for the MS1 signal of the heavy spike-in trigger peptide. Since this SIL peptide is chemically identical to the endogenous (that is, light) peptide, the spiked-in and the endogenous peptides have almost (note that deuterated peptides can show a significant RT shift) identical elution profiles. Therefore, the presence of the trigger peptide reliably indicates the RT at which the targeted peptide is also eluting, even in case its abundance is too low to be directly detected in watch mode. Detection of the trigger peptide then initiates the quantification mode that involves sensitive scanning for the light target peptide at the MS2 and/or MS3 level with long dwell times. Depending on the specific implementation of the method, quantitative scans per target may take up to 5 s.

Spike-in-triggered acquisition was originally published as “index-ion Triggered MS2 Ion Quantification” (iMSTIQ) (58) or “Internal Standard Triggered-Parallel Reaction Monitoring” (IS-PRM) (59) and has been further developed since then. Current implementations of this concept mainly differ in the specific acquisition algorithms employed by the mass spectrometer. For example, a potential problem of spike-in-triggered acquisition is that MS1 background peaks with m/z ratios corresponding to trigger peptides can erroneously initiate the quantification mode, which leads to loss of valuable measurement time. In two of the methods mentioned below (*SureQuant* and *TOMAHAQ*), the quantification scans are only triggered after an MS2-level “spectrum check” of the trigger peptide as an additional filter to enhance selectivity.

Spike-in-triggered acquisition methods have the advantage that the presence of the trigger peptides precisely shows where the endogenous target peptides are expected to be found, facilitating their detection. In addition, SIL trigger peptides can serve as a reliable internal reference for relative (and potentially absolute) quantification. The main disadvantage of these methods is that selecting, producing, and validating suitable trigger peptides are time-consuming and costly.

### SureQuant and Related Technologies

SureQuant was developed by Thermo and is preinstalled on the Orbitrap Exploris 480 and Orbitrap Eclipse tribrid systems. In a SureQuant run, detection of spiked-in SIL peptides over a predefined intensity threshold prompts a fast low-resolution MS2 scan of the trigger peptides. The presence of trigger peptides is then confirmed *via* pseudo-spectral matching against preselected product ions. A positive match initiates a highly sensitive quantitative scan for the target peptide. The dwell time can be set individually for each target peptide. In addition to serving as a trigger, the spiked-in SIL peptides can also be used as a reference for precise quantification by monitoring both the heavy spiked-in and light endogenous peptides throughout their elution period. For example, similar to SRM methods (60, 61), a recent paper described the targeted analysis of 340 tyrosine-phosphorylated peptides across 31 colorectal cancer tumors (62). In total, 91% of the SIL and 78% of the endogenous phosphopeptides could be detected. While this study required synthesis of a custom set of SIL trigger peptides, assay kits for certain pathways or protein sets (AKT/mTOR pathway, PQ500 for serum/plasma) are commercially available.

SureQuant requires specific algorithms in the acquisition software that are currently only available on the specific newer Thermo instruments. For other Orbitrap-type instruments, Pseudo-PRM might provide an alternative means of implementing spike-in triggered acquisition (63). The method utilizes the offset fragmentation options in the acquisition software. The mass spectrometer operates in DDA mode with SIL-trigger peptides added to an inclusion list. Detection of the trigger peptide in an MS1 scan then initiates a targeted scan with a mass offset corresponding to the m/z ratio of the endogenous peptide, leading to continuous fragmentation of the target peptide. This simple acquisition method does not employ a “spectrum check” to reduce erroneous triggering. Further benchmarking experiments to investigate the false-positive rate in triggered scans would increase credibility of the method.

### TOMAHAQ

TOMAHAQ (triggered-by-offset, multiplexed, accurate-mass, high-resolution, and absolute quantification) is a spike-in-triggered targeting method that takes advantage of isobaric labeling-based multiplexing (64, 65). The different samples to be compared with each other are labeled using tandem mass tags (TMT) (66) and combined. In parallel, synthetic trigger peptides are labeled with an alternative TMT reagent termed TMTsh (TMT superheavy) that coelutes with the corresponding target peptide at an offset m/z ratio. Detection of TMTsh synthetic trigger peptides sets off a series of scans to verify and identify the trigger peptide, identify the target peptide, optimize the quantification scan, and finally an optimized MS3 scan for target TMT reporter ions with dwell times as long as 5 s. Quantification at the MS3 level is advantageous because it reduces coisolation interference, which would otherwise impair TMT reporter ion-based quantification—a pervasive problem especially in complex samples (67). TOMAHAQ enabled targeting of 520 peptides in unfractionated “single shot” samples, and the data obtained correlated well with DDA data from fractionated samples.

Tomahto (64), the software to run TOMAHAQ acquisition methods, is freely available and compatible with Tribrid instruments. It only requires a list of peptide targets, which greatly simplifies setting up methods. The key advantage of TOMAHAQ/Tomahto is that TMT-based multiplexing greatly increases the number of samples that can be analyzed per unit time. Furthermore, the spectral match to confirm the presence of the trigger peptide, as well as the long accumulation of TMT reporter ions (if necessary), makes the method very sensitive. It is, however, more complex than *SureQuant* or *Pseudo-PRM* and may also be affected by the TMT inherent quantification biases.

### Scout-MRM

Instead of SIL peptides, Scout-MRM is based on so-called “scout” peptides to trigger targeted acquisition (68, 69). These scout peptides are designed to span the entire RT range and do not correspond to target peptides. To set up targeted acquisition, target peptides are associated with one or several scout peptides that have similar retention times. During targeted acquisition, the detection of scout peptides triggers complex acquisition schemes for all associated target peptides. An advantage of this method is that trigger peptides are universal and do not need to be obtained for each target. However, since scout peptides do not have the same amino acid sequences as target peptides, they are less reliable than spiked-in SIL peptides for detection and quantification. Scout-MRM is currently only available on Sciex instruments. The recently released Peptide selector (69) tool simplifies the design of Scout-MRM methods.

## Conclusion

Advanced targeted proteomics methods are emerging as attractive alternatives to untargeted DDA and DIA approaches. Smarter experimental designs and/or more advanced data acquisition algorithms enable sensitive detection of a higher number of targets per run. While computational proteomics so far mostly focused on the post acquisition analysis of mass spectrometric data, an emerging trend is to also use advanced algorithms before and during data acquisition. Optimizing the data acquisition process using intelligent scheduling approaches allows users to better take advantage of the analytical power of the mass spectrometers employed. Also, these methods have been used to target entire signaling pathways and thus provide users with information about specific features they are interested in.

The development and adaptation of the advanced acquisition methods would benefit from standardized technical benchmarking experiments that enable a comparison within the advanced acquisition methods themselves and with the conventional targeted acquisition methods. For example, for the *RT adjustment based methods*, the accuracy of the RT prediction for different chromatographic setups is relevant. For *spike-in free RT adjustment methods*, the robustness against variation in background should be shown. For the *spike-in triggered acquisition methods*, factors such as reproducible triggering of the targeted scans and the number of scans acquired across the LC peak with increasing numbers of targets would be interesting to compare between the advanced methods, but also with conventional methods. Additionally, validation procedures similar to the ones established for conventional targeting methods (34) support potential users in choosing the right strategy for the project.

When should advanced acquisition proteomics method be used, and which method is the best fit? The answer to this question depends on a number of factors. First, the analytical challenge of the project is important: A simple targeting method, like PRM, can reproducibly detect a few rather abundant peptides, whereas monitoring hundreds of low abundance peptides is more challenging. SRM methods are sensitive and able to quantify a large number targets (Table 1), but selecting appropriate transitions is tedious and time-consuming, making the assay hard to design or customize. For applications such as the quantification of signaling pathway activity, mapping of downstream biomarkers or cross-validation experiments to verify DDA and DIA results, the targets need to be quickly customizable and one of the advanced acquisition strategies might be a good fit. A second factor relates to the number of samples to be measured and the ease of implementing the methods: On the one hand, *Picky* is a simple tool that allows for quick customization of composition of target peptides, and therefore well suited for project-specific orthogonal cross-validation experiments and arguably preferable over western blotting (70). On the other hand, methods such as *TOMAHAQ* and *SureQuant* benefit from the improved run-to-run reproducibility, increased number of targets, sensitivity of spike-in-triggered acquisition, and sample multiplexing (for *TOMAHAQ*). However, the need for custom trigger peptides makes these methods uneconomical for projects involving a small number of samples. *iRT-based methods*, *spike-in free RT adjustment methods,* and *Scout-MRM* are somewhere in the middle between these two poles. A third and often decisive factor is simply the availability of a method on a certain mass spectrometer. For example, *TOMAHAQ* is only available on Thermo Tribrid instruments, and *Scout-MRM* has only been implemented on Sciex machines.

The ease of implementing these advanced targeted acquisitions also depends on the availability of method design tools. Picky, TOMAHAQ and Scout-MRM come with software to select proteotypic peptides and plan their scheduled acquisition. To this end, Picky and Peptide selector (the tool associated with Scout-MRM) connect to the databases ProteomeTools (24) and SRMatlas (23), respectively. Peptide iRT values are also integrated in databases and in tools such as Skyline (27).

In the future, we predict that targeted proteomics will assert itself as an attractive alternative to global DDA and DIA approaches. On the one hand, we expect that smart experimental designs in combination with sophisticated acquisition algorithms will be able to extract more and more useful information from samples in less and less time. This will also be supported by further improvements on the hardware side, such as increased MS scan rates, or using ion mobility for additional fractionation in the gas phase (71). Additionally, there are many other directions of improvements in the field of targeted proteomics that are outside the scope of this review. For example, the further expansion of online databases with empirical data such as PeptideAtlas (72), ProteomicsDB (24, 73) and Phosphopedia (74) for phosphopeptides can aid in the selection of detectable and specific proteotypic peptides and fragment ions. In a similar fashion, databases with targeted proteomic-specific data such as SRMatlas (23) and the CPTAC Assay Portal (75) can aid in the adoption of targeted methods. The continued development of tools that further simplify access to these databases (such as Skyline or Picky), especially by integrating information from these public repositories, facilitates the design of targeted methods. Other efforts have focused on increasing sensitivity by improving sample preparation such as enrichment or “offline” separation techniques (5, 38, 76). Antibody-based immuno-affinity enrichment or immuno-SRM focuses on enrichment of target peptides using antibodies early before sample preparation. SISCAPA (Stable Isotope Standards and Capture by Anti-Peptide Antibodies) (77, 78, 79) combines antibody-based enrichment with stable isotope-labeled quantification. The target peptides and heavy-labeled “counterparts” are captured by the antibodies simultaneously before MS analysis. Application of the method depends on the availability of antibodies (80). The method is widely applied, for example, to study plasma (79), phospho-signaling (18, 81) and the biological activity of Thalidomide analogs (82). The combination of antibody-based enrichment with advanced targeted acquisition methods would enable further improvement of the sensitivity of targeted proteomics.

Furthermore, the continued improvements of data-processing methods and tools, such as data analysis with Skyline and embedded tools (27, 83, 84) and Spectrodive, are especially important with increasing number of targets and large number of samples. Such developments will facilitate research by providing detailed functional information, for example, the activity of specific pathways (62, 64). On the other hand, less complex but more robust targeted acquisition approaches will likely become more relevant in clinical settings and could complement antibody-based detection for diagnosis and stratification (85, 86, 87).

## Conflict of interest

The authors declare no competing interests.
